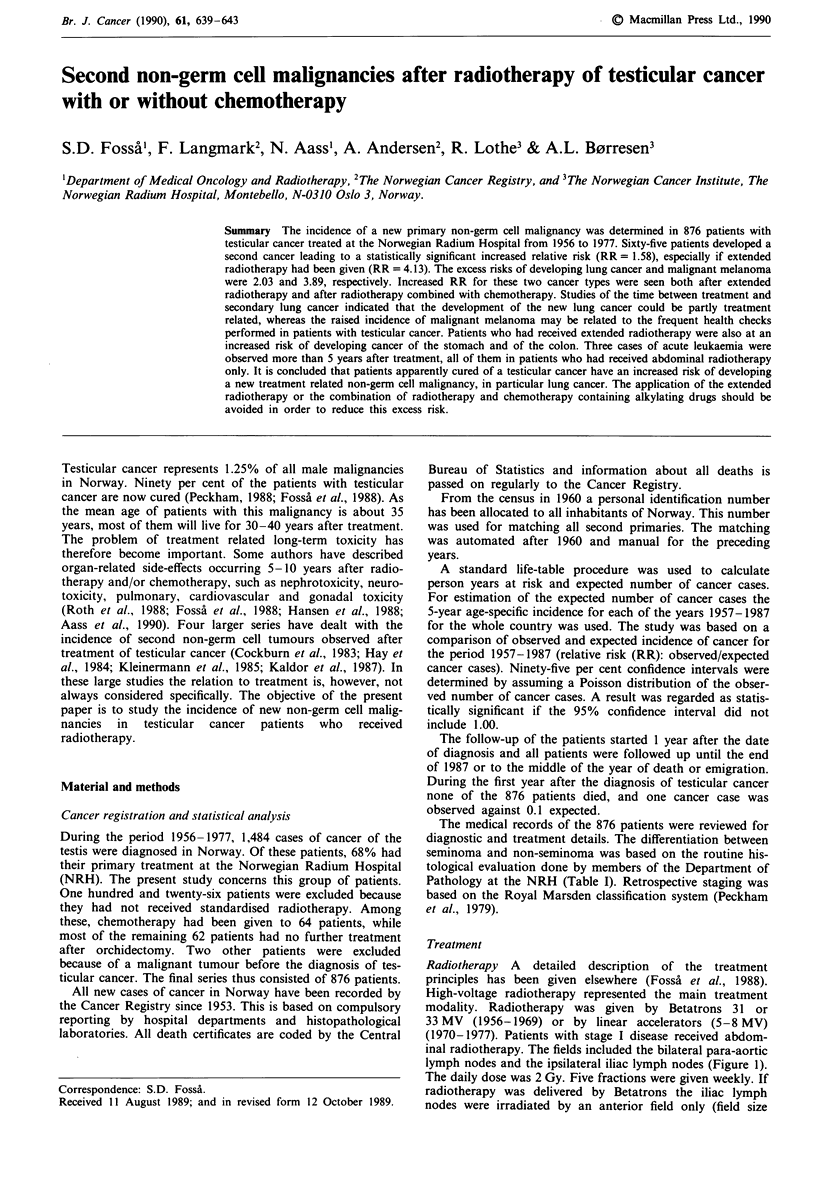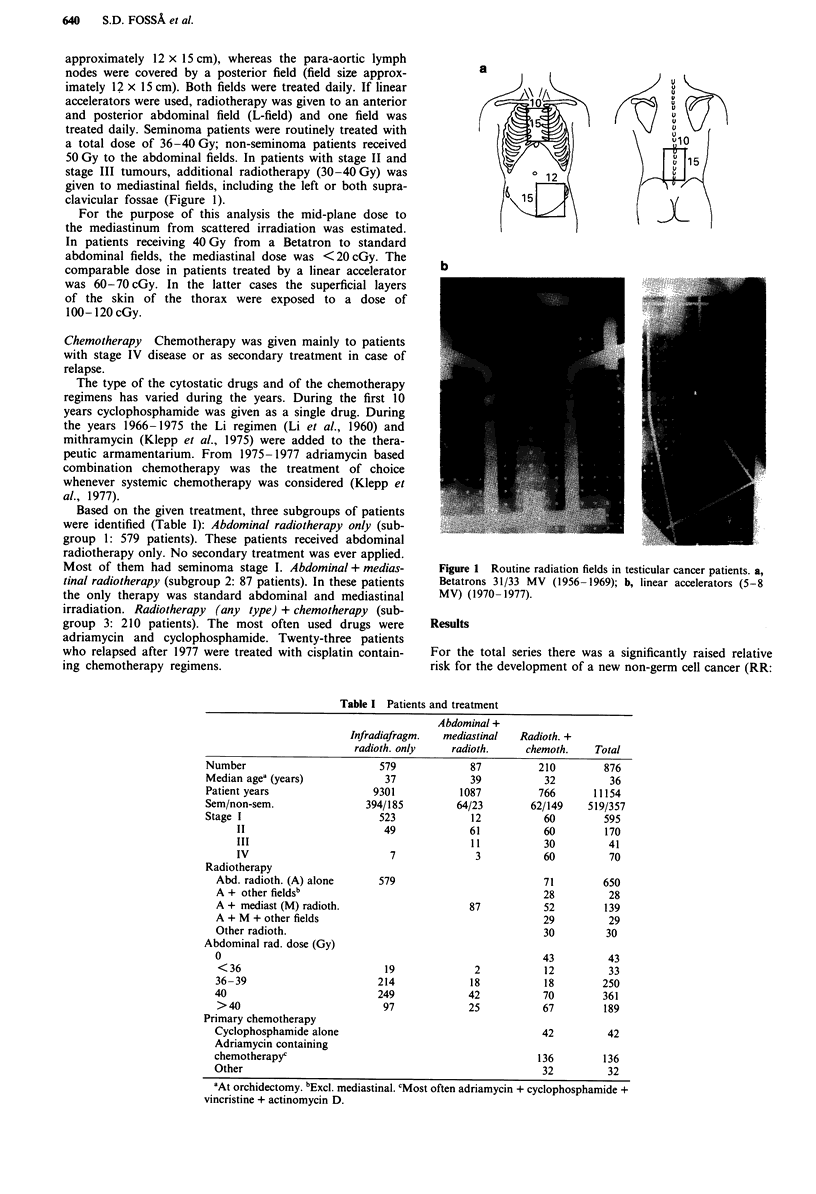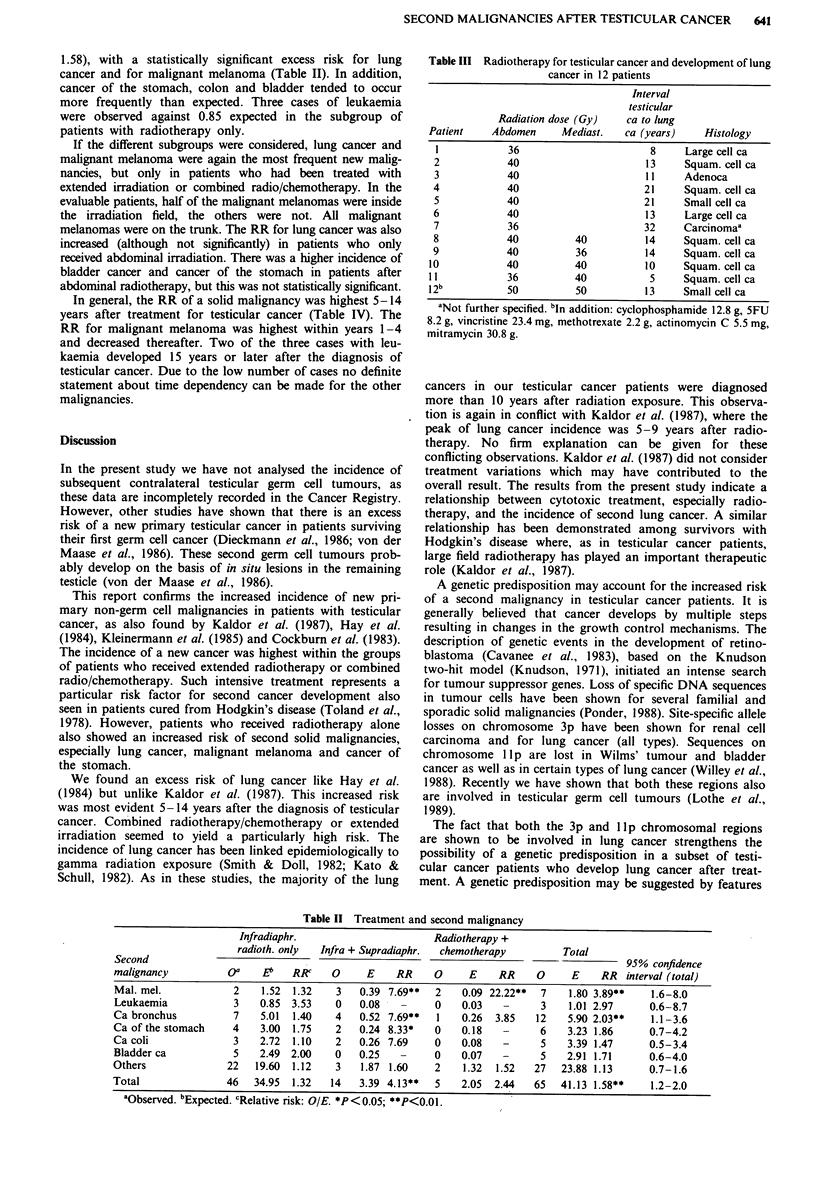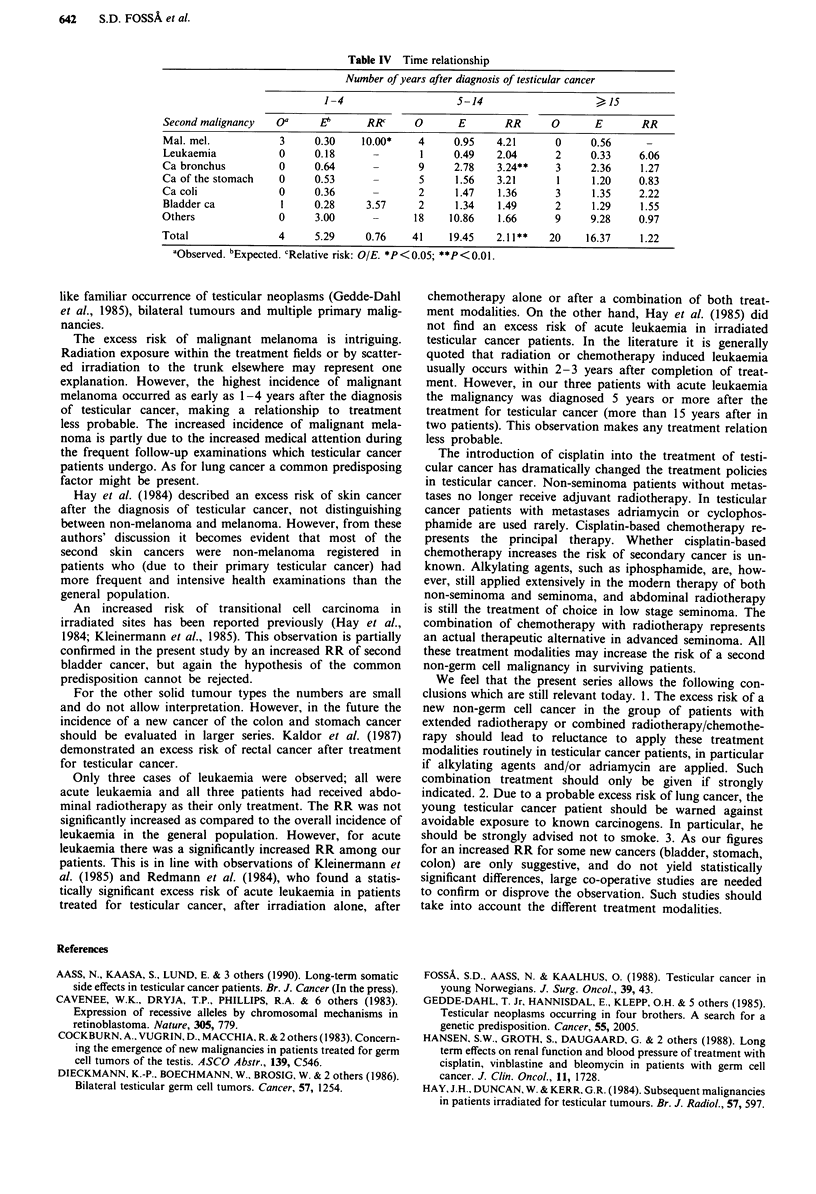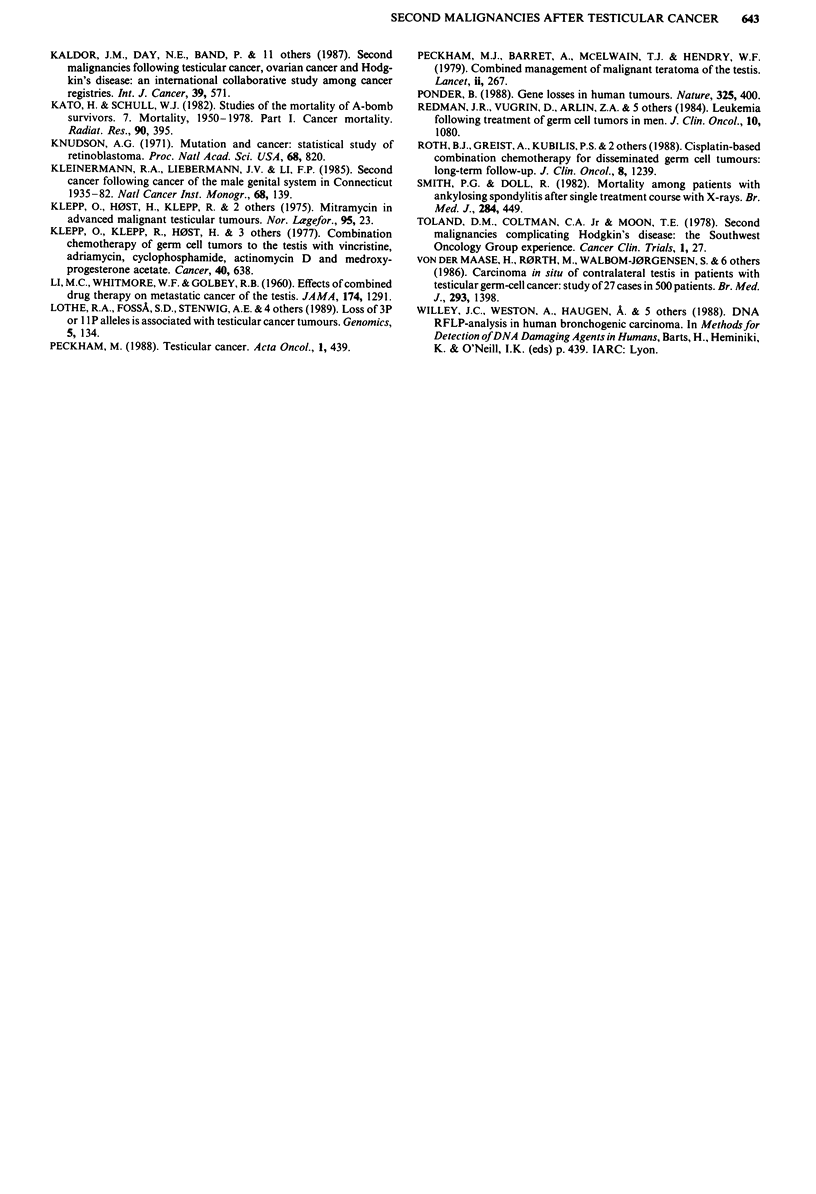# Second non-germ cell malignancies after radiotherapy of testicular cancer with or without chemotherapy.

**DOI:** 10.1038/bjc.1990.142

**Published:** 1990-04

**Authors:** S. D. Fosså, F. Langmark, N. Aass, A. Andersen, R. Lothe, A. L. Børresen

**Affiliations:** Department of Medical Oncology and Radiotherapy, Norwegian Radium Hospital, Montebello, Oslo.

## Abstract

**Images:**


Br. J. Cancer (1990), 61, 639-643 © Macmillan Press Ltd., 1990

### Second non-germ cell malignancies after radiotherapy of testicular cancer with or without chemotherapy

S.D. Fosså[1], F. Langmark[2], N. Aass[1], A. Andersen[2], R. Lothe[3] & A.L. Børresen[3]

[1]*Department of Medical Oncology and Radiotherapy,* [2]*The Norwegian Cancer Registry, and* [3]*The Norwegian Cancer Institute, The Norwegian Radium Hospital, Montebello, N-0310 Oslo 3, Norway.*

**Summary** The incidence of a new primary non-germ cell malignancy was determined in 876 patients with testicular cancer treated at the Norwegian Radium Hospital from 1956 to 1977. Sixty-five patients developed a second cancer leading to a statistically significant increased relative risk (RR = 1.58), especially if extended radiotherapy had been given (RR = 4.13). The excess risks of developing lung cancer and malignant melanoma were 2.03 and 3.89, respectively. Increased RR for these two cancer types were seen both after extended radiotherapy and after radiotherapy combined with chemotherapy. Studies of the time between treatment and secondary lung cancer indicated that the development of the new lung cancer could be partly treatment related, whereas the raised incidence of malignant melanoma may be related to the frequent health checks performed in patients with testicular cancer. Patients who had received extended radiotherapy were also at an increased risk of developing cancer of the stomach and of the colon. Three cases of acute leukaemia were observed more than 5 years after treatment, all of them in patients who had received abdominal radiotherapy only. It is concluded that patients apparently cured of a testicular cancer have an increased risk of developing a new treatment related non-germ cell malignancy, in particular lung cancer. The application of the extended radiotherapy or the combination of radiotherapy and chemotherapy containing alkylating drugs should be avoided in order to reduce this excess risk.

Testicular cancer represents 1.25% of all male malignancies in Norway. Ninety per cent of the patients with testicular cancer are now cured (Peckham, 1988; Fosså *et al.*, 1988). As the mean age of patients with this malignancy is about 35 years, most of them will live for 30-40 years after treatment. The problem of treatment related long-term toxicity has therefore become important. Some authors have described organ-related side-effects occurring 5-10 years after radiotherapy and/or chemotherapy, such as nephrotoxicity, neurotoxicity, pulmonary, cardiovascular and gonadal toxicity (Roth *et al.*, 1988; Fosså *et al.*, 1988; Hansen *et al.*, 1988; Aass *et al.*, 1990). Four larger series have dealt with the incidence of second non-germ cell tumours observed after treatment of testicular cancer (Cockburn *et al.*, 1983; Hay *et al.*, 1984; Kleinermann *et al.*, 1985; Kaldor *et al.*, 1987). In these large studies the relation to treatment is, however, not always considered specifically. The objective of the present paper is to study the incidence of new non-germ cell malignancies in testicular cancer patients who received radiotherapy.

### Material and methods

### *Cancer registration and statistical analysis*

During the period 1956-1977, 1,484 cases of cancer of the testis were diagnosed in Norway. Of these patients, 68% had their primary treatment at the Norwegian Radium Hospital (NRH). The present study concerns this group of patients. One hundred and twenty-six patients were excluded because they had not received standardised radiotherapy. Among these, chemotherapy had been given to 64 patients, while most of the remaining 62 patients had no further treatment after orchidectomy. Two other patients were excluded because of a malignant tumour before the diagnosis of testicular cancer. The final series thus consisted of 876 patients.

All new cases of cancer in Norway have been recorded by the Cancer Registry since 1953. This is based on compulsory reporting by hospital departments and histopathological laboratories. All death certificates are coded by the Central Bureau of Statistics and information about all deaths is passed on regularly to the Cancer Registry.

From the census in 1960 a personal identification number has been allocated to all inhabitants of Norway. This number was used for matching all second primaries. The matching was automated after 1960 and manual for the preceding years.

A standard life-table procedure was used to calculate person years at risk and expected number of cancer cases. For estimation of the expected number of cancer cases the 5-year age-specific incidence for each of the years 1957-1987 for the whole country was used. The study was based on a comparison of observed and expected incidence of cancer for the period 1957-1987 (relative risk (RR): observed/expected cancer cases). Ninety-five per cent confidence intervals were determined by assuming a Poisson distribution of the observed number of cancer cases. A result was regarded as statistically significant if the 95% confidence interval did not include 1.00.

The follow-up of the patients started 1 year after the date of diagnosis and all patients were followed up until the end of 1987 or to the middle of the year of death or emigration. During the first year after the diagnosis of testicular cancer none of the 876 patients died, and one cancer case was observed against 0.1 expected.

The medical records of the 876 patients were reviewed for diagnostic and treatment details. The differentiation between seminoma and non-seminoma was based on the routine histological evaluation done by members of the Department of Pathology at the NRH (Table I). Retrospective staging was based on the Royal Marsden classification system (Peckham *et al.*, 1979).

### *Treatment*

*Radiotherapy* A detailed description of the treatment principles has been given elsewhere (Fosså *et al.*, 1988). High-voltage radiotherapy represented the main treatment modality. Radiotherapy was given by Betatrons 31 or 33 MV (1956-1969) or by linear accelerators (5-8 MV) (1970-1977). Patients with stage I disease received abdominal radiotherapy. The fields included the bilateral para-aortic lymph nodes and the ipsilateral iliac lymph nodes (Figure 1). The daily dose was 2 Gy. Five fractions were given weekly. If radiotherapy was delivered by Betatrons the iliac lymph nodes were irradiated by an anterior field only (field size

Correspondence: S.D. Fosså.
Received 11 August 1989; and in revised form 12 October 1989.

approximately 12 × 15 cm), whereas the para-aortic lymph nodes were covered by a posterior field (field size approximately 12 × 15 cm). Both fields were treated daily. If linear accelerators were used, radiotherapy was given to an anterior and posterior abdominal field (L-field) and one field was treated daily. Seminoma patients were routinely treated with a total dose of 36–40 Gy; non-seminoma patients received 50 Gy to the abdominal fields. In patients with stage II and stage III tumours, additional radiotherapy (30–40 Gy) was given to mediastinal fields, including the left or both supraclavicular fossae (Figure 1).

For the purpose of this analysis the mid-plane dose to the mediastinum from scattered irradiation was estimated. In patients receiving 40 Gy from a Betatron to standard abdominal fields, the mediastinal dose was <20 cGy. The comparable dose in patients treated by a linear accelerator was 60–70 cGy. In the latter cases the superficial layers of the skin of the thorax were exposed to a dose of 100–120 cGy.

*Chemotherapy* Chemotherapy was given mainly to patients with stage IV disease or as secondary treatment in case of relapse.

The type of the cytostatic drugs and of the chemotherapy regimens has varied during the years. During the first 10 years cyclophosphamide was given as a single drug. During the years 1966–1975 the Li regimen (Li *et al.*, 1960) and mithramycin (Klepp *et al.*, 1975) were added to the therapeutic armamentarium. From 1975–1977 adriamycin based combination chemotherapy was the treatment of choice whenever systemic chemotherapy was considered (Klepp *et al.*, 1977).

Based on the given treatment, three subgroups of patients were identified (Table I): *Abdominal radiotherapy only* (subgroup 1: 579 patients). These patients received abdominal radiotherapy only. No secondary treatment was ever applied. Most of them had seminoma stage I. *Abdominal + mediastinal radiotherapy* (subgroup 2: 87 patients). In these patients the only therapy was standard abdominal and mediastinal irradiation. *Radiotherapy (any type) + chemotherapy* (subgroup 3: 210 patients). The most often used drugs were adriamycin and cyclophosphamide. Twenty-three patients who relapsed after 1977 were treated with cisplatin containing chemotherapy regimens.

**Figure 1** Routine radiation fields in testicular cancer patients. **a**, Betatrons 31/33 MV (1956–1969); **b**, linear accelerators (5–8 MV) (1970–1977).

### Results

For the total series there was a significantly raised relative risk for the development of a new non-germ cell cancer (RR:

**Table I** Patients and treatment

| | *Infradiafragm. radioth. only* | *Abdominal + mediastinal radioth.* | *Radioth. + chemoth.* | *Total* |
|---|---|---|---|---|
| Number | 579 | 87 | 210 | 876 |
| Median age[a] (years) | 37 | 39 | 32 | 36 |
| Patient years | 9301 | 1087 | 766 | 11154 |
| Sem/non-sem. | 394/185 | 64/23 | 62/149 | 519/357 |
| Stage I | 523 | 12 | 60 | 595 |
| II | 49 | 61 | 60 | 170 |
| III | | 11 | 30 | 41 |
| IV | 7 | 3 | 60 | 70 |
| Radiotherapy | | | | |
| Abd. radioth. (A) alone | 579 | | 71 | 650 |
| A + other fields[b] | | | 28 | 28 |
| A + mediast (M) radioth. | | 87 | 52 | 139 |
| A + M + other fields | | | 29 | 29 |
| Other radioth. | | | 30 | 30 |
| Abdominal rad. dose (Gy) | | | | |
| 0 | | | 43 | 43 |
| <36 | 19 | 2 | 12 | 33 |
| 36–39 | 214 | 18 | 18 | 250 |
| 40 | 249 | 42 | 70 | 361 |
| >40 | 97 | 25 | 67 | 189 |
| Primary chemotherapy | | | | |
| Cyclophosphamide alone | | | 42 | 42 |
| Adriamycin containing chemotherapy[c] | | | 136 | 136 |
| Other | | | 32 | 32 |

[a]At orchidectomy. [b]Excl. mediastinal. [c]Most often adriamycin + cyclophosphamide + vincristine + actinomycin D.

1.58), with a statistically significant excess risk for lung cancer and for malignant melanoma (Table II). In addition, cancer of the stomach, colon and bladder tended to occur more frequently than expected. Three cases of leukaemia were observed against 0.85 expected in the subgroup of patients with radiotherapy only.

If the different subgroups were considered, lung cancer and malignant melanoma were again the most frequent new malignancies, but only in patients who had been treated with extended irradiation or combined radio/chemotherapy. In the evaluable patients, half of the malignant melanomas were inside the irradiation field, the others were not. All malignant melanomas were on the trunk. The RR for lung cancer was also increased (although not significantly) in patients who only received abdominal irradiation. There was a higher incidence of bladder cancer and cancer of the stomach in patients after abdominal radiotherapy, but this was not statistically significant.

In general, the RR of a solid malignancy was highest 5–14 years after treatment for testicular cancer (Table IV). The RR for malignant melanoma was highest within years 1–4 and decreased thereafter. Two of the three cases with leukaemia developed 15 years or later after the diagnosis of testicular cancer. Due to the low number of cases no definite statement about time dependency can be made for the other malignancies.

### Discussion

In the present study we have not analysed the incidence of subsequent contralateral testicular germ cell tumours, as these data are incompletely recorded in the Cancer Registry. However, other studies have shown that there is an excess risk of a new primary testicular cancer in patients surviving their first germ cell cancer (Dieckmann *et al.*, 1986; von der Maase *et al.*, 1986). These second germ cell tumours probably develop on the basis of *in situ* lesions in the remaining testicle (von der Maase *et al.*, 1986).

This report confirms the increased incidence of new primary non-germ cell malignancies in patients with testicular cancer, as also found by Kaldor *et al.* (1987), Hay *et al.* (1984), Kleinermann *et al.* (1985) and Cockburn *et al.* (1983). The incidence of a new cancer was highest within the groups of patients who received extended radiotherapy or combined radio/chemotherapy. Such intensive treatment represents a particular risk factor for second cancer development also seen in patients cured from Hodgkin's disease (Toland *et al.*, 1978). However, patients who received radiotherapy alone also showed an increased risk of second solid malignancies, especially lung cancer, malignant melanoma and cancer of the stomach.

We found an excess risk of lung cancer like Hay *et al.* (1984) but unlike Kaldor *et al.* (1987). This increased risk was most evident 5–14 years after the diagnosis of testicular cancer. Combined radiotherapy/chemotherapy or extended irradiation seemed to yield a particularly high risk. The incidence of lung cancer has been linked epidemiologically to gamma radiation exposure (Smith & Doll, 1982; Kato & Schull, 1982). As in these studies, the majority of the lung cancers in our testicular cancer patients were diagnosed more than 10 years after radiation exposure. This observation is again in conflict with Kaldor *et al.* (1987), where the peak of lung cancer incidence was 5–9 years after radiotherapy. No firm explanation can be given for these conflicting observations. Kaldor *et al.* (1987) did not consider treatment variations which may have contributed to the overall result. The results from the present study indicate a relationship between cytotoxic treatment, especially radiotherapy, and the incidence of second lung cancer. A similar relationship has been demonstrated among survivors with Hodgkin's disease where, as in testicular cancer patients, large field radiotherapy has played an important therapeutic role (Kaldor *et al.*, 1987).

A genetic predisposition may account for the increased risk of a second malignancy in testicular cancer patients. It is generally believed that cancer develops by multiple steps resulting in changes in the growth control mechanisms. The description of genetic events in the development of retinoblastoma (Cavanee *et al.*, 1983), based on the Knudson two-hit model (Knudson, 1971), initiated an intense search for tumour suppressor genes. Loss of specific DNA sequences in tumour cells have been shown for several familial and sporadic solid malignancies (Ponder, 1988). Site-specific allele losses on chromosome 3p have been shown for renal cell carcinoma and for lung cancer (all types). Sequences on chromosome 11p are lost in Wilms' tumour and bladder cancer as well as in certain types of lung cancer (Willey *et al.*, 1988). Recently we have shown that both these regions also are involved in testicular germ cell tumours (Lothe *et al.*, 1989).

The fact that both the 3p and 11p chromosomal regions are shown to be involved in lung cancer strengthens the possibility of a genetic predisposition in a subset of testicular cancer patients who develop lung cancer after treatment. A genetic predisposition may be suggested by features

**Table III** Radiotherapy for testicular cancer and development of lung cancer in 12 patients

| *Patient* | *Radiation dose (Gy)* *Abdomen* | *Mediast.* | *Interval testicular ca to lung ca (years)* | *Histology* |
|---|---|---|---|---|
| 1 | 36 | | 8 | Large cell ca |
| 2 | 40 | | 13 | Squam. cell ca |
| 3 | 40 | | 11 | Adenoca |
| 4 | 40 | | 21 | Squam. cell ca |
| 5 | 40 | | 21 | Small cell ca |
| 6 | 40 | | 13 | Large cell ca |
| 7 | 36 | | 32 | Carcinoma[a] |
| 8 | 40 | 40 | 14 | Squam. cell ca |
| 9 | 40 | 36 | 14 | Squam. cell ca |
| 10 | 40 | 40 | 10 | Squam. cell ca |
| 11 | 36 | 40 | 5 | Squam. cell ca |
| 12[b] | 50 | 50 | 13 | Small cell ca |

[a]Not further specified. [b]In addition: cyclophosphamide 12.8 g, 5FU 8.2 g, vincristine 23.4 mg, methotrexate 2.2 g, actinomycin C 5.5 mg, mitramycin 30.8 g.

**Table II** Treatment and second malignancy

| *Second malignancy* | *Infradiaphr. radioth. only* $O^a$ | $E^b$ | $RR^c$ | *Infra + Supradiaphr.* *O* | *E* | *RR* | *Radiotherapy + chemotherapy* *O* | *E* | *RR* | *Total* *O* | *E* | *RR* | *95% confidence interval (total)* |
|---|---|---|---|---|---|---|---|---|---|---|---|---|---|
| Mal. mel. | 2 | 1.52 | 1.32 | 3 | 0.39 | 7.69** | 2 | 0.09 | 22.22** | 7 | 1.80 | 3.89** | 1.6–8.0 |
| Leukaemia | 3 | 0.85 | 3.53 | 0 | 0.08 | – | 0 | 0.03 | – | 3 | 1.01 | 2.97 | 0.6–8.7 |
| Ca bronchus | 7 | 5.01 | 1.40 | 4 | 0.52 | 7.69** | 1 | 0.26 | 3.85 | 12 | 5.90 | 2.03** | 1.1–3.6 |
| Ca of the stomach | 4 | 3.00 | 1.75 | 2 | 0.24 | 8.33* | 0 | 0.18 | – | 6 | 3.23 | 1.86 | 0.7–4.2 |
| Ca coli | 3 | 2.72 | 1.10 | 2 | 0.26 | 7.69 | 0 | 0.08 | – | 5 | 3.39 | 1.47 | 0.5–3.4 |
| Bladder ca | 5 | 2.49 | 2.00 | 0 | 0.25 | – | 0 | 0.07 | – | 5 | 2.91 | 1.71 | 0.6–4.0 |
| Others | 22 | 19.60 | 1.12 | 3 | 1.87 | 1.60 | 2 | 1.32 | 1.52 | 27 | 23.88 | 1.13 | 0.7–1.6 |
| Total | 46 | 34.95 | 1.32 | 14 | 3.39 | 4.13** | 5 | 2.05 | 2.44 | 65 | 41.13 | 1.58** | 1.2–2.0 |

[a]Observed. [b]Expected. [c]Relative risk: $O/E$. *$P<0.05$; **$P<0.01$.

**Table IV** Time relationship

| | Number of years after diagnosis of testicular cancer | | | | | | | | |
|---|---|---|---|---|---|---|---|---|---|
| | 1–4 | | | 5–14 | | | ≥15 | | |
| Second malignancy | O[a] | E[b] | RR[c] | O | E | RR | O | E | RR |
| Mal. mel. | 3 | 0.30 | 10.00* | 4 | 0.95 | 4.21 | 0 | 0.56 | – |
| Leukaemia | 0 | 0.18 | – | 1 | 0.49 | 2.04 | 2 | 0.33 | 6.06 |
| Ca bronchus | 0 | 0.64 | – | 9 | 2.78 | 3.24** | 3 | 2.36 | 1.27 |
| Ca of the stomach | 0 | 0.53 | – | 5 | 1.56 | 3.21 | 1 | 1.20 | 0.83 |
| Ca coli | 0 | 0.36 | – | 2 | 1.47 | 1.36 | 3 | 1.35 | 2.22 |
| Bladder ca | 1 | 0.28 | 3.57 | 2 | 1.34 | 1.49 | 2 | 1.29 | 1.55 |
| Others | 0 | 3.00 | – | 18 | 10.86 | 1.66 | 9 | 9.28 | 0.97 |
| Total | 4 | 5.29 | 0.76 | 41 | 19.45 | 2.11** | 20 | 16.37 | 1.22 |

[a]Observed. [b]Expected. [c]Relative risk: $O/E$. $*P<0.05$; $**P<0.01$.

like familiar occurrence of testicular neoplasms (Gedde-Dahl *et al.*, 1985), bilateral tumours and multiple primary malignancies.

The excess risk of malignant melanoma is intriguing. Radiation exposure within the treatment fields or by scattered irradiation to the trunk elsewhere may represent one explanation. However, the highest incidence of malignant melanoma occurred as early as 1–4 years after the diagnosis of testicular cancer, making a relationship to treatment less probable. The increased incidence of malignant melanoma is partly due to the increased medical attention during the frequent follow-up examinations which testicular cancer patients undergo. As for lung cancer a common predisposing factor might be present.

Hay *et al.* (1984) described an excess risk of skin cancer after the diagnosis of testicular cancer, not distinguishing between non-melanoma and melanoma. However, from these authors' discussion it becomes evident that most of the second skin cancers were non-melanoma registered in patients who (due to their primary testicular cancer) had more frequent and intensive health examinations than the general population.

An increased risk of transitional cell carcinoma in irradiated sites has been reported previously (Hay *et al.*, 1984; Kleinermann *et al.*, 1985). This observation is partially confirmed in the present study by an increased RR of second bladder cancer, but again the hypothesis of the common predisposition cannot be rejected.

For the other solid tumour types the numbers are small and do not allow interpretation. However, in the future the incidence of a new cancer of the colon and stomach cancer should be evaluated in larger series. Kaldor *et al.* (1987) demonstrated an excess risk of rectal cancer after treatment for testicular cancer.

Only three cases of leukaemia were observed; all were acute leukaemia and all three patients had received abdominal radiotherapy as their only treatment. The RR was not significantly increased as compared to the overall incidence of leukaemia in the general population. However, for acute leukaemia there was a significantly increased RR among our patients. This is in line with observations of Kleinermann *et al.* (1985) and Redmann *et al.* (1984), who found a statistically significant excess risk of acute leukaemia in patients treated for testicular cancer, after irradiation alone, after chemotherapy alone or after a combination of both treatment modalities. On the other hand, Hay *et al.* (1985) did not find an excess risk of acute leukaemia in irradiated testicular cancer patients. In the literature it is generally quoted that radiation or chemotherapy induced leukaemia usually occurs within 2–3 years after completion of treatment. However, in our three patients with acute leukaemia the malignancy was diagnosed 5 years or more after the treatment for testicular cancer (more than 15 years after in two patients). This observation makes any treatment relation less probable.

The introduction of cisplatin into the treatment of testicular cancer has dramatically changed the treatment policies in testicular cancer. Non-seminoma patients without metastases no longer receive adjuvant radiotherapy. In testicular cancer patients with metastases adriamycin or cyclophosphamide are used rarely. Cisplatin-based chemotherapy represents the principal therapy. Whether cisplatin-based chemotherapy increases the risk of secondary cancer is unknown. Alkylating agents, such as iphosphamide, are, however, still applied extensively in the modern therapy of both non-seminoma and seminoma, and abdominal radiotherapy is still the treatment of choice in low stage seminoma. The combination of chemotherapy with radiotherapy represents an actual therapeutic alternative in advanced seminoma. All these treatment modalities may increase the risk of a second non-germ cell malignancy in surviving patients.

We feel that the present series allows the following conclusions which are still relevant today. 1. The excess risk of a new non-germ cell cancer in the group of patients with extended radiotherapy or combined radiotherapy/chemotherapy should lead to reluctance to apply these treatment modalities routinely in testicular cancer patients, in particular if alkylating agents and/or adriamycin are applied. Such combination treatment should only be given if strongly indicated. 2. Due to a probable excess risk of lung cancer, the young testicular cancer patient should be warned against avoidable exposure to known carcinogens. In particular, he should be strongly advised not to smoke. 3. As our figures for an increased RR for some new cancers (bladder, stomach, colon) are only suggestive, and do not yield statistically significant differences, large co-operative studies are needed to confirm or disprove the observation. Such studies should take into account the different treatment modalities.

### References

AASS, N., KAASA, S., LUND, E. & 3 others (1990). Long-term somatic side effects in testicular cancer patients. *Br. J. Cancer* (In the press).

CAVENEE, W.K., DRYJA, T.P., PHILLIPS, R.A. & 6 others (1983). Expression of recessive alleles by chromosomal mechanisms in retinoblastoma. *Nature*, **305**, 779.

COCKBURN, A., VUGRIN, D., MACCHIA, R. & 2 others (1983). Concerning the emergence of new malignancies in patients treated for germ cell tumors of the testis. *ASCO Abstr.*, **139**, C546.

DIECKMANN, K.-P., BOECHMANN, W., BROSIG, W. & 2 others (1986). Bilateral testicular germ cell tumors. *Cancer*, **57**, 1254.

FOSSÅ, S.D., AASS, N. & KAALHUS, O. (1988). Testicular cancer in young Norwegians. *J. Surg. Oncol.*, **39**, 43.

GEDDE-DAHL, T. Jr, HANNISDAL, E., KLEPP, O.H. & 5 others (1985). Testicular neoplasms occurring in four brothers. A search for a genetic predisposition. *Cancer*, **55**, 2005.

HANSEN, S.W., GROTH, S., DAUGAARD, G. & 2 others (1988). Long term effects on renal function and blood pressure of treatment with cisplatin, vinblastine and bleomycin in patients with germ cell cancer. *J. Clin. Oncol.*, **11**, 1728.

HAY, J.H., DUNCAN, W. & KERR, G.R. (1984). Subsequent malignancies in patients irradiated for testicular tumours. *Br. J. Radiol.*, **57**, 597.

KALDOR, J.M., DAY, N.E., BAND, P. & 11 others (1987). Second malignancies following testicular cancer, ovarian cancer and Hodgkin's disease: an international collaborative study among cancer registries. *Int. J. Cancer*, **39**, 571.

KATO, H. & SCHULL, W.J. (1982). Studies of the mortality of A-bomb survivors. 7. Mortality, 1950–1978. Part I. Cancer mortality. *Radiat. Res.*, **90**, 395.

KNUDSON, A.G. (1971). Mutation and cancer: statistical study of retinoblastoma. *Proc. Natl Acad. Sci. USA*, **68**, 820.

KLEINERMANN, R.A., LIEBERMANN, J.V. & LI, F.P. (1985). Second cancer following cancer of the male genital system in Connecticut 1935–82. *Natl Cancer Inst. Monogr.*, **68**, 139.

KLEPP, O., HØST, H., KLEPP, R. & 2 others (1975). Mitramycin in advanced malignant testicular tumours. *Nor. Lægefor.*, **95**, 23.

KLEPP, O., KLEPP, R., HØST, H. & 3 others (1977). Combination chemotherapy of germ cell tumors to the testis with vincristine, adriamycin, cyclophosphamide, actinomycin D and medroxyprogesterone acetate. *Cancer*, **40**, 638.

LI, M.C., WHITMORE, W.F. & GOLBEY, R.B. (1960). Effects of combined drug therapy on metastatic cancer of the testis. *JAMA*, **174**, 1291.

LOTHE, R.A., FOSSÅ, S.D., STENWIG, A.E. & 4 others (1989). Loss of 3P or 11P alleles is associated with testicular cancer tumours. *Genomics*, **5**, 134.

PECKHAM, M. (1988). Testicular cancer. *Acta Oncol.*, **1**, 439.

PECKHAM, M.J., BARRET, A., MCELWAIN, T.J. & HENDRY, W.F. (1979). Combined management of malignant teratoma of the testis. *Lancet*, **ii**, 267.

PONDER, B. (1988). Gene losses in human tumours. *Nature*, **325**, 400.

REDMAN, J.R., VUGRIN, D., ARLIN, Z.A. & 5 others (1984). Leukemia following treatment of germ cell tumors in men. *J. Clin. Oncol.*, **10**, 1080.

ROTH, B.J., GREIST, A., KUBILIS, P.S. & 2 others (1988). Cisplatin-based combination chemotherapy for disseminated germ cell tumours: long-term follow-up. *J. Clin. Oncol.*, **8**, 1239.

SMITH, P.G. & DOLL, R. (1982). Mortality among patients with ankylosing spondylitis after single treatment course with X-rays. *Br. Med. J.*, **284**, 449.

TOLAND, D.M., COLTMAN, C.A. Jr & MOON, T.E. (1978). Second malignancies complicating Hodgkin's disease: the Southwest Oncology Group experience. *Cancer Clin. Trials*, **1**, 27.

VON DER MAASE, H., RØRTH, M., WALBOM-JØRGENSEN, S. & 6 others (1986). Carcinoma *in situ* of contralateral testis in patients with testicular germ-cell cancer: study of 27 cases in 500 patients. *Br. Med. J.*, **293**, 1398.

WILLEY, J.C., WESTON, A., HAUGEN, Å. & 5 others (1988). DNA RFLP-analysis in human bronchogenic carcinoma. In *Methods for Detection of DNA Damaging Agents in Humans*, Barts, H., Heminiki, K. & O'Neill, I.K. (eds) p. 439. IARC: Lyon.